# Crosstalk between ferroptosis and chondrocytes in osteoarthritis: a systematic review of *in vivo* and *in vitro* studies

**DOI:** 10.3389/fimmu.2023.1202436

**Published:** 2023-07-14

**Authors:** Siyang Cao, Yihao Wei, Huihui Xu, Jian Weng, Tiantian Qi, Fei Yu, Su Liu, Ao Xiong, Peng Liu, Hui Zeng

**Affiliations:** ^1^ Department of Bone & Joint Surgery, Peking University Shenzhen Hospital, Shenzhen, Guangdong, China; ^2^ National & Local Joint Engineering Research Centre of Orthopaedic Biomaterials, Peking University Shenzhen Hospital, Shenzhen, Guangdong, China; ^3^ Shenzhen Key Laboratory of Orthopaedic Diseases and Biomaterials Research, Peking University Shenzhen Hospital, Shenzhen, Guangdong, China

**Keywords:** ferroptosis, chondrocytes, osteoarthritis, crosstalk, systematic review

## Abstract

**Purpose:**

Recent scientific reports have revealed a close association between ferroptosis and the occurrence and development of osteoarthritis (OA). Nevertheless, the precise mechanisms by which ferroptosis influences OA and how to hobble OA progression by inhibiting chondrocyte ferroptosis have not yet been fully elucidated. This study aims to conduct a comprehensive systematic review (SR) to address these gaps.

**Methods:**

Following the guidelines of the Preferred Reporting Items for Systematic Reviews and Meta-Analyses (PRISMA) 2020, we conducted a comprehensive search of the Embase, Ovid, ProQuest, PubMed, Scopus, the Cochrane Library, and Web of Science databases to identify relevant studies that investigate the association between ferroptosis and chondrocytes in OA. Our search included studies published from the inception of these databases until January 31st, 2023. Only studies that met the predetermined quality criteria were included in this SR.

**Results:**

In this comprehensive SR, a total of 21 studies that met the specified criteria were considered suitable and included in the current updated synthesis. The mechanisms underlying chondrocyte ferroptosis and its association with OA progression involve various biological phenomena, including mitochondrial dysfunction, dysregulated iron metabolism, oxidative stress, and crucial signaling pathways.

**Conclusion:**

Ferroptosis in chondrocytes has opened an entirely new chapter for the investigation of OA, and targeted regulation of it is springing up as an attractive and promising therapeutic tactic for OA.

**Systematic review registration:**

https://inplasy.com/inplasy-2023-3-0044/, identifier INPLASY202330044.

## 1 Introduction

Osteoarthritis (OA) is the most common form of arthritis (1), affecting 7% of the global human population (2). With the aged tendency of population and higher rates of obesity, the incidence of OA is expected to proliferate (3), which will have a mounting and unavoidable impact on and major challenges for global health care and each country’s public health system. For a considerable period, OA has been perceived as a degenerative ailment resulting from mechanical stress. However, there are indications that the inflammation observed in OA is chronic, of a relatively low intensity, and primarily mediated by the innate immune system (4). Due to the intricate and complex onset of OA, its etiology and underlying molecular or inflammatory immune mechanisms remain inadequately elucidated.

Notably, earlier research revealed that cartilage degeneration plays a salient role in the progression of OA (5), while the evolution of OA is associated with oxidative stress and reactive oxygen species (ROS) (6, 7). Both the engendering of ROS and the consequent lipid peroxidation are bound up with the antioxidant capabilities of chondrocytes and occupy significant places within cartilage degeneration (8, 9). Meanwhile, the breakdown of iron homeostasis and hoard of surplus iron in tissues are linked with oxidative stress, which may cause chondrocytes’ injury and damage cartilage homeostasis (10, 11). As such, it is of significant interest to probe the role of iron and ROS in the advancement of OA.

Ferroptosis, a novel form of nonapoptotic cell death characterised by the iron-dependent accumulation of lipid hydroperoxides (12), has garnered growing attention over the past decade (13). Recent research has indicated that ferroptosis may participate in immunity, thereby contributing to the regulation of inflammatory damage, signal transduction, and cellular proliferation (14). Based on the aforementioned associations between OA and innate immunity, as well as between ferroptosis and innate immunity, it can be deduced that ferroptosis potentially exerts a significant influence on the etiology and advancement of OA. The ferroptosis of chondrocytes that sparks the progression of OA was initially authenticated by Yao et al. in 2021 (15), and a contemporaneous paper by Jing et al. attested that iron dyshomeostasis is associated with the accelerated progression of OA (16). Shortly afterwards, studies of OA related to chondrocyte ferroptosis began to mushroom, and this has continued over the last two years.

To gain a better understanding of the nexus between ferroptosis and chondrocytes in OA, and to proffer novel insights and unseal a new orientation for in-depth research in both pre-clinical and clinical settings, a rigorous and robust systematic review (SR) is warranted. Based upon the summary of up-to-date *in vivo* and *in vitro* research advances, this SR is expected to lay a firm and solid groundwork for future researchers in the realm of OA-ferroptosis. To the best of our knowledge, no SRs concerning ferroptosis and chondrocytes in OA have been published thus far.

## 2 Materials and methods

### 2.1 Registration and protocol

This SR was enrolled on the International Platform of Registered Systematic Review and Meta-analysis Protocols. Its registration code is INPLASY202330044, and the protocol can be found at https://inplasy.com/inplasy-2023-3-0044/. The present SR abides by the Preferred Reporting Items for Systematic Reviews and Meta-Analyses (PRISMA) 2020 guidelines (17).

### 2.2 Search strategy

A systematic search of Embase, Ovid, ProQuest, PubMed, Scopus, the Cochrane Library and Web of Science was carried out from the inception of the respective databases up to January 31st, 2023, and used the following medical subject heading terms and free words: (‘ferroptosis’ OR ‘iron death’ OR ‘iron overload’) AND (‘osteoarthritis’ OR ‘osteoarthritides’ OR ‘osteoarthrosis’ OR ‘degenerative arthritis’ OR ‘arthroses’ OR ‘osteoarthrosis deformans’). Detailed information about the retrieval strategy can be found in the **Supplementary Materials**.

### 2.3 Eligibility criteria

The inclusion criteria were (1) study investigated chondrocytes’ ferroptosis in OA (2) containing cell and/or animal experiments (3) in the English-language literature. The exclusion criteria were (1) review papers, dissertations, letters, commentaries, editorials, conference abstracts, meta-analyses, clinical trials, case reports or bioinformatics analysis, (2) the same studies published in different journals under the same or different titles and (3) full text that was inaccessible.

### 2.4 Study selection

Studies collected from the initial search were imported into NoteExpress version 3.7 (ANGEAN SEA Technology, Beijing, China) to organise the related literature and eliminate duplicate references. The final eligibility of the retrieved papers was determined by two adjudicators (SYC and HHX), who independently scrutinised the titles and abstracts of the papers. Discrepancies were addressed via consensus with a third reviewer (SL).

### 2.5 Risk of bias assessment

The *in vivo* studies’ risk of bias evaluation was performed independently by two researchers (SYC and YHW) using Review Manager 5.3 (Cochrane Collaboration, Oxford, UK), according to the Systematic Review Centre for Laboratory Animal Experimentation’s (SYRCLE) risk of bias tool (18). For cellular experiments, two of the abovementioned authors independently assessed the bias risk table for chondrocyte experiments adapted from previous studies (**Table 1**) (19). All discrepancies were resolved by discussion and adjudication by a third researcher (HHX).

**Table 1:** Domains and descriptions for the appraisal of the risk of bias for cell experiments.

Item	Type of bias (Domain)	Description of domain	Review authors’ judgment
1	Selection bias (Selection of chondrocytes)	The selection of chondrocyte cells should be performed from commercially available cell lines or from cartilage samples collected from animals or human patients with consent. In both cases, chondrocytes should be obtained from hyaline cartilage. Control and intervention groups should be clearly defined.	Studies isolating cells from more than one anatomical site were judged to have a high risk of bias.
2	Selection bias (Confounding variables)	Chondrocytes should be isolated from more than one animal with the same characteristics (type, race, weight and age) from the same anatomical site. Cartilage should be collected from the same anatomical sites, and isolated chondrocytes should have the same viability and count among groups. Studies should implement the same isolation protocol and the same protocol for establishing the primary cell culture(s). The number of cell passages should be the same for all experimental groups and should not be too high, since chondrocytes lose their phenotype with an increasing number of passages. The same experimental conditions and cell density should be guaranteed for both the control and intervention groups.	The studies that did not report the cell density or animal characteristics from which the cells were extracted were judged to have an unclear risk of bias.
3	Performance bias (Exposure measurement)	Measurement techniques should be adequate and well-established for the specific outcomes that the studies are assessing, and their measurement protocol should be clearly described to allow for replication. Semi-quantitative and/or qualitative analysis should be performed by two independent observers to ascertain inter-operator reliability.	Studies that did not employ two independent observers for qualitative or semi-qualitative analysis were judged to have a high risk of bias.
4	Detection bias (Blinding outcome assessment)	The outcome assessor and/or data analyst was not blinded to groups (i.e. intervention vs. control). For quantitative analyses, the blinding of the outcome assessor and/or data analyst was not considered necessary. Otherwise (in semi-quantitative and qualitative analyses), blinding was required.	If no information about performing the semi-quantitative analysis blindly was provided, the studies were judged to have a risk of bias.
5	Attrition bias (Incomplete outcome data)	Missing data in > 5% of outcome variables.	Studies failed to provide any information on the number of replicas were judged to have an unclear risk of bias.
6	Reporting bias (Selective outcome reporting)	Based on reporting of the collected/assessed outcomes and multiple subgroup analyses.	Studies failed to show the results for all the outcomes measured or for experimental and control groups were judged to have a high risk of bias.
7	Other (Funding bias)	Conflict of interest by the study authors and/or industry sponsorship.	Studies failed to provide any information on conflict of interests were judged to have an unclear risk of bias.

### 2.6 Data collection and extraction

Information from *in vivo* and *in vitro* studies was synthesised narratively and reported using a standardised data extraction form. The following data were collected: author (year), country, cell type and source, animal species, animal age, weight and gender, sample size, core study design, drug delivery approach, duration of intervention, outcome measures and pivotal discovery. One reviewer (SYC) extracted the data, and another (YHW) independently checked their accuracy. Team consensus was sought to resolve any discrepancies.

## 3 Results and discussion

### 3.1 Study selection

**Figure 1:**
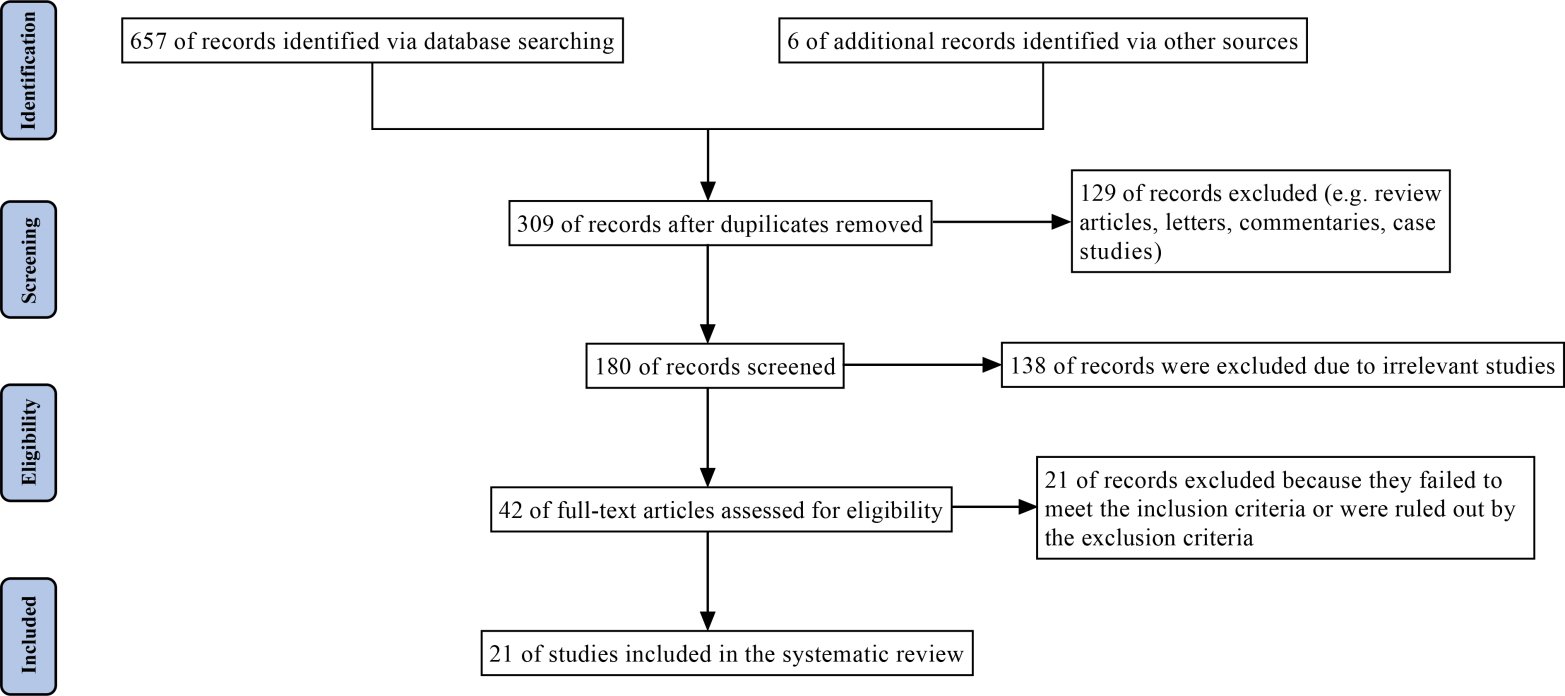
PRISMA flow diagram of the systematic literature search and selection.

In total, 657 records were identified after a comprehensive search of seven databases; these included 142 records in Embase, 67 in Ovid, 92 in ProQuest, 37 in PubMed, 117 in Scopus, two in the Cochrane Library and 200 in Web of Science. Six records were incorporated via other sources in parallel. After 354 duplicates were removed, 267 studies were excluded by title and abstract. The full texts of the 42 remaining articles were assessed, and a further 21 studies were eliminated because they failed to meet the inclusion criteria or attained the exclusion criteria. Twenty-one studies were ultimately selected in this up-to-date SR. For more detail, please refer to **Figure 1** and the **Supplementary Materials**.

### 3.2 Study characteristics

**Figure 2:**
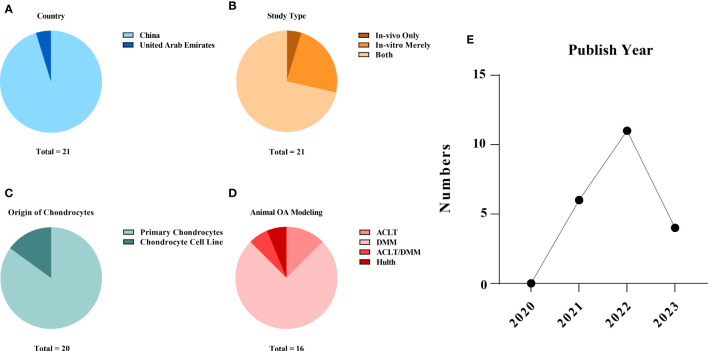
Study characteristics of the systematic review: **(A)** publication origin, **(B)** categories of experiments, **(C)** the sources of the chondrocytes applied in the studies, **(D)** animal modelling of the *in vivo* studies and **(E)** the year of publication of the selected studies. The data cut-off date of the present analysis was January 31st, 2023.

The studies were all from within the past three years (2020~2023), with over half of them (11/21, 52.38%) published in 2022. Twenty publications (95.23%) were from China (15, 16, 20–37), and one other (4.77%) was from the United Arab Emirates (38). *In vitro* and *in vivo* experiments were performed simultaneously in 15 (71.43%) of the 21 studies (15, 16, 20–25, 27–29, 33–35, 37). Cellular-only experiments were carried out in five (23.81%) studies (26, 30, 31, 36, 38). A team from China conducted solely a validation of animal experiments (1/21, 4.77%) (32). An overview of the embodied studies is shown in **Figure 2**. The primary features (cell model, animal model, ferroptotic detection means etc.) of the incorporated studies are encapsulated in **Tables 2**, **3**.

**Table 2:** Characteristics of the *in vitro* studies.

Author (year, Country)	Cell type & source	Core study design (Duration of intervention)	Outcome measures	Pivotal discovery
Gong et al. (27) (2023, China)	Primary chondrocytes (healthy); SD rat (knee joint)	G1: control G2: IL-1β (10 ng/mL) G3: IL-1β (10 ng/mL) + CAD (10 μmol/L) (24 hours)	**Cell viability assay:** CCK-8 kit **Intracellular iron level:** iron assay kit **Intracellular GSH level:** GSH test kit **Intracellular ROS and lipid ROS level:** DCFH-DA and C11-BODIPY **Malondialdehyde assay:** MDA assay kit **Mitochondrial membrane potential:** JC-1 staining **Mitochondrial morphology:** Mito-Tracker **Morphology:** TB and Alcian Blue staining **Protein expression:** WB and IF **Ultrastructure of mitochondria:** TEM	CAD ameliorated OA cartilage degradation by regulating ferroptosis via the p53/SLC7A11/GPx4 signalling pathway.
Guo et al. (29) (2022, China)	Primary chondrocytes (healthy); C57BL/6J mice (knee joint)	G1: control G2: IL-1β (10 ng/mL) G3: IL-1β (10 ng/mL) + DFO (100 μmol/L) G4: IL-1β (10 ng/mL) + DFO (100 μmol/L) + si-Nrf2 (24 hours)	**Cell viability assay:** CCK-8 kit **Intracellular iron level:** iron assay kit **Intracellular ROS and lipid ROS level:** DCFH-DA and C11-BODIPY **Malondialdehyde assay:** MDA assay kit **Morphology:** TB staining **Protein expression:** WB **Ultrastructure of mitochondria:** TEM	DFO alleviated the inflammatory response and ECM degradation in chondrocytes induced by IL-1β by inhibiting chondrocyte ferroptosis via Nrf2 signalling.
He et al. (24) (2023, China)	Primary chondrocytes (healthy); C57BL/6J mice (knee joint)	G1: control G2: FAC (500 μmol/L) G3: FAC (500 μmol/L) + DFO (100 μmol/L) G4: FAC (500 μmol/L) + BCA (12 μmol/L) G5: FAC (500 μmol/L) + BCA (24 μmol/L) (48 hours)	**Apoptotic effect:** annexin V-FITC/PI staining **Cell viability assay:** CCK-8 kit **Gene quantification:** PCR **Intracellular iron level:** calcein-AM **Intracellular ROS and lipid ROS level:** DCFH-DA and C11-BODIPY **Intracellular GSH:** GSH test kit **Mitochondrial membrane potential:** JC-1 staining **Morphology:** TB staining **Protein expression:** WB	Biochanin A protects against iron overload–associated knee osteoarthritis by regulating iron levels and the Nrf2 / System Xc^-^ / GPx4 axis
Jing et al. (16) (2021, China) a	Primary chondrocytes (healthy); C57BL/6J mice (knee joint)	G1: control G2: IL-1β (10 ng/mL) G3: TNF-α (10 ng/mL) G4: IL-1β (10 ng/mL) + si-DMT1 G5: TNF-α (10 ng/mL) + si-DMT1 (24 hours)	**Apoptotic effect:** annexin V-FITC/PI staining and flow cytometry **Gene quantification:** PCR **Intracellular iron level:** calcein-AM **Protein expression:** WB	DMT1 played a pivotal role in iron overload–induced OA progress. Inhibition of DMT1 suppressed IL-1β-induced inflammatory response and ECM degradation by blockading MAPK and PI3K/AKT pathway.
Jing et al. (30) (2021, China)b	Primary chondrocytes (healthy); C57BL/6J mice (knee joint)	G1: control G2: FAC (100 μmol/L) G3: FAC (100 μmol/L) + IL-1β (10 ng/mL) G4: FAC (100 μmol/L) + DFO (100 μmol/L) G5: FAC (100 μmol/L) + NAC (100 μmol/L) (24 hours)	**Apoptotic effect:** annexin V-FITC/PI staining **Gene quantification:** PCR **Intracellular iron level:** calcein-AM **Intracellular ROS level:** ROS assay kit **Mitochondrial membrane potential:** JC-1 staining and flow cytometer **Mitochondrial morphology:** Mito-Tracker **Protein expression:** WB	The pro-inflammatory cytokines could disrupt chondrocytes’ iron homeostasis and promote iron influx; iron overload–induced oxidative stress and mitochondrial dysfunction play important roles in iron overload–induced cartilage degeneration.
Jing et al. (26) (2021 China)c	Primary chondrocytes (healthy); C57BL/6J mice (knee joint)	G1: control G2: FAC (100 μmol/L) G3: FAC (100 μmol/L) + BA (10 μmol/L) (24 hours)	**Apoptotic effect:** annexin V-FITC/PI staining **Cell viability assay:** CCK-8 kit **Gene quantification:** PCR **Intracellular iron level:** calcein-AM **Intracellular ROS level:** ROS assay kit **Mitochondrial membrane potential:** JC-1 staining **Mitochondrial morphology:** Mito-Tracker **Protein expression:** WB and IF	Calcium chelators may be of value in the treatment of iron metabolism–related diseases and IOOA progression.
Karim et al. (38) (2022, United Arab Emirates)	Immortalised C-20/A4 cell line; human	G1: control G2: FAC (200 μmol/L) G3: FAC (300 μmol/L) (24/48 hours)	**Apoptotic effect:** annexin V-FITC/PI staining **Cell cycle progression analysis:** flow cytometer **Cell viability assay:** MTT kit **Intracellular iron level:** calcein-AM and flow cytometer **Intracellular ROS level:** ROS assay kit **Protein expression:** WB and IF	Iron overload disrupts cellular iron homeostasis, which compromises the functional integrity of chondrocytes and leads to oxidative stress and apoptosis.
Liu et al. (31) (2022, China)	Primary chondrocytes (OA); human (articular cartilage)	G1: control G2: sh-SLC3A2-1 G3: sh-SLC3A2-2 G4: sh-SLC3A2-1 + Fer-1 (unknown drug concentration) G5: sh-SLC3A2-2 + Fer-1 (unknown drug concentration) (24/48/72 hours)	**Gene quantification:** PCR **Morphology:** Alcian Blue, SOFG and TB staining **Protein expression:** WB **Ultrastructure of mitochondria:** TEM	SLC3A2 inhibited ferroptosis and suppressed cartilage degeneration in OA.
Lv M et al. (33) (2022, China)	Primary chondrocytes (healthy); mice (unknown race, knee joint)	G1: control G2: IL-1β (10 ng/mL) G3: IL-1β (10 ng/mL) + sh-SND1 G4: IL-1β (10 ng/mL) + sh-SND1 + sh-HSPA5 G5: IL-1β (10 ng/mL) + Ad-HSPA5 G6: IL-1β (10 ng/mL) + Ad-HSPA5 +sh-GPx4 (48 hours)	**Cell viability assay:** MTT kit and EdU staining **Intracellular iron level:** ELISA kit **Intracellular ROS and lipid ROS level:** DCFH-DA **Malondialdehyde assay:** ELISA kit **RNA-binding protein interaction:** RIP **Protein expression:** CO-IP and WB	The RNA-binding protein SND1 promotes the degradation of GPx4 by destabilising the HSPA5 mRNA and suppressing HSPA5 expression, promoting ferroptosis in osteoarthritis chondrocytes.
Lv Z et al. (35) (2022, China)	Primary chondrocytes (healthy); C57BL/6J mice (knee joint)	G1: control G2: CPS (50 μmol/L) G3: JNJ (10 μmol/L) G4: TBHP (50 μmol/L) G5: CPS (50 μmol/L) + TBHP (50 μmol/L) G6: JNJ (50 μmol/L) +TBHP (50 μmol/L) (24 hours)	**Cell viability assay:** CCK-8 kit and AM/PI staining **Gene quantification:** PCR **Intracellular iron level:** FerroOrange **Intracellular ROS and lipid ROS level:** DCFH-DA and C11-BODIPY **Protein expression:** WB	TRPV1 protects chondrocytes from ferroptosis.
Miao et al. (28) (2022, China)	ATDC5 cell line; mouse	G1: sh-NC + IL-1β (10 ng/mL) G2: sh-GPx4-1 + IL-1β (10 ng/mL) G3: sh-GPx4-2 + IL-1β (10 ng/mL) (NR)	**Cell viability assay:** CCK-8 kit **Gene quantification:** PCR **Intracellular GSH:** GSH test kit **Intracellular iron level:** FerroOrange **Intracellular ROS and lipid ROS level:** DCFH-DA and Liperfluo staining **Malondialdehyde assay:** MDA assay kit **Mitochondrial membrane potential:** JC-1 staining **Protein expression:** WB	GPx4 downregulation could increase the sensitivity of chondrocytes to oxidative stress and aggravate ECM degradation through the MAPK/NF-κB pathway.
Mo et al. (36) (2021, China)	ATDC5 cell line; mouse	G1: control G2: IL-1β (10 ng/mL) G3: IL-1β (10 ng/mL) + STM (20 μg/mL) G4: IL-1β (10 ng/mL) + STM (20 μg/mL) + Oe-NC G5: IL-1β (10 ng/mL) + STM (20 μg/mL) + Oe-SREBF2 (24 hours)	**Analysis of oxidative stress:** GSH, MDA and SOD assay kit **Cell viability assay:** CCK-8 kit **Gene quantification:** PCR **Intracellular iron level:** iron colorimetric assay kit **Protein expression:** WB	STM attenuated chondrocyte injury induced by IL-1β by regulating ferroptosis via down-regulation of SREBF2 and may have potential as a novel therapeutic method for knee osteoarthritis.
Pan et al. (21) (2022, China)	Primary chondrocytes (healthy); C57BL/6J mice (knee joint)	G1: control G2: FAC (100 μmol/L) + IL-1β (10 ng/mL) G3: FAC (100 μmol/L) + IL-1β (10 ng/mL) + NAC (100 μmol/L) G4: FAC (100 μmol/L) + IL-1β (10 ng/mL) + NAR-L (10 μmol/L) G5: FAC (100 μmol/L) + IL-1β (10 ng/mL) + NAR-H (20 μmol/L) (48 hours)	**Apoptotic effect:** annexin V-FITC/PI staining and flow cytometry **Cell viability assay:** CCK-8 kit **Gene quantification:** PCR **Intracellular iron level:** calcein-AM **Intracellular ROS and lipid ROS level:** DCFH-DA and C11-BODIPY **Malondialdehyde assay:** MDA assay kit **Mitochondrial membrane potential:** JC-1 staining and flow cytometer **Morphology:** TB staining **Protein expression:** WB	NAR can reduce oxidative stress through the Nrf2/HO-1 pathway and alleviate cartilage damage under iron overload and has the potential to treat IOOA.
Wan et al. (25) (2023, China)	1. Primary chondrocytes (healthy); human (articular cartilage) 2. Primary chondrocytes (healthy); mice (unknown race, knee joint) 3. AMPKα-knockout mice	G1: control G2: IL-1β (10 ng/mL) G3: AICAR (10 mmol/L) + IL-1β (10 ng/mL) G4: IL-1β (10 ng/mL) + baicalein (5 μmol/L) G5: AICAR (10 mmol/L) + IL-1β (10 ng/mL) + baicalein (5 μmol/L) (36 hours)	**Apoptotic effect:** annexin V-FITC/PI staining and flow cytometry **Cell proliferation assay:** EdU staining **Cell viability assay:** CCK-8 kit **Gene quantification:** PCR **Intracellular iron level:** Phen green SK diacetate **Intracellular lipid ROS level:** C11-BODIPY **Protein expression:** WB and Co-IP **Ultrastructure of mitochondria:** TEM	Baicalein suppresses ferroptosis by inducing AMPK phosphorylation and facilitating AMPK holoenzyme assembly, stability and activity.
Wang S et al. (23) (2022, China)	Primary chondrocytes; Col2a1-CreERT GPx4^flox/flox^ mice (knee joint)	G1: control G2: 1 MPa (1 Hz) G3: 1 MPa (1 Hz) + GsMTx4 (10 nmol/L) (24 hours)	**Cell live/dead assay:** calcein/PI cell viability/cytotoxicity assay kit **Gene quantification:** PCR **Intracellular GSH level:** GSH assay kit **Intracellular ROS level:** DCFH-DA **Mitochondrial membrane potential:** JC-1 staining **Mitochondrial morphology:** Mito-Tracker **Protein expression:** WB **Ultrastructure of mitochondria:** TEM	Mechanical overloading induced ferroptosis in chondrocytes through the Piezo 1 ion channel.
Wang X et al. (22) (2022, China)	Primary chondrocytes (healthy); SD rat (knee joint)	G1: control G2: IL-1β (10 ng/mL) G3: IL-1β (10 ng/mL) + Fer-1 (1 μmol/L) G4: IL-1β (10 ng/mL) + ATX (10 μmol/L) (24 hours)	**Cell viability assay:** CCK-8 kit **Intracellular GSH level:** GSH test kit **Intracellular iron level:** colorimetric assay **Intracellular ROS and lipid ROS level:** DCFH-DA and C11-BODIPY **Malondialdehyde assay:** MDA assay kit **Mitochondrial iron level:** Mito-FerroGreen **Mitochondrial membrane potential:** JC-1 staining **Mitochondrial morphology:** Mito-Tracker **Morphology:** TB staining **Protein expression level:** WB and IF **Ultrastructure of mitochondria:** TEM	Both Fer-1 and ATX are able to mitigate chondrocyte injury and osteoarthritis progression by inhibiting ferroptosis and the regulation of mitochondrial function.
Wen et al. (34) (2023, China)	Primary chondrocytes (OA); human (articular cartilage)	G1: control G2: UCPH-101 (20 μmol/L) G3: siRNA EAAT1 G4: siRNA control (24 hours)	**Apoptotic effect:** PI/Hoechst **Cell viability assay:** CCK-8 kit **Gene quantification:** PCR **Intracellular GSH and GSSG level:** GSH and GSSG test kit **Intracellular iron level:** iron assay kit **Intracellular ROS and lipid ROS level:** DCFH-DA and C11-BODIPY **Malondialdehyde assay:** MDA assay kit **Ratio of senescent:** β-galactosidase staining **Protein expression:** WB	The EAAT1-glutamate-GPx4 anti-ferroptosis axis is a key survival mechanism for SenChos, and EAAT1 is an effective and specific target for anti-senescence therapy in osteoarthritis.
Xu et al. (37) (2022, China)	Primary chondrocytes (OA); human (articular cartilage)	G1: si-NC G2: erastin (5 μmol/L) + si-NC G3: erastin (5 μmol/L) + si-NC + TF3 (30 μmol/L) G4: erastin (5 μmol/L) + si-Nrf2 + TF3 (30 μmol/L) (24 hours)	**Apoptotic effect:** PI staining **Cytotoxicity assays:** MTS assay kit **Gene quantification:** PCR **Intracellular ROS level:** DCFH-DA **Mitochondrial iron level:** Mito-FerroGreen **Protein expression level:** WB	TF3 significantly inhibits chondrocyte ferroptosis by activating the Nrf2/GPx4 signalling pathway, suggesting that TF3 serves as a potential therapeutic supplement for OA.
Yao et al. (15) (2021, China)	Primary chondrocytes (healthy); C57BL/6J mice (knee joint)	G1: control G2: IL-1β (10 ng/mL) G3: IL-1β (10 ng/mL) + Fer-1 (1 μmol/L) G4: FAC (100 μmol/L) G5: FAC (100 μmol/L) + Fer-1 (1 μmol/L) (48 hours)	**Cell viability assay:** CCK-8 kit **Intracellular ROS and lipid ROS level:** DCFH-DA and C11-BODIPY **Protein expression level:** WB and IF	Chondrocyte ferroptosis contributes to the progression of osteoarthritis.
Zhou et al. (20) (2021,China)	Primary chondrocytes (healthy); C57BL/6J mice (knee joint)	G1: mock G2: mock + IL-1β (10 ng/mL) G3: mock + IL-1β (10 ng/mL) + D-mannose G4: Ad-Epas1 + IL-1β (10 ng/mL) G5: Ad-Epas1 + IL-1β (10 ng/mL) + D-mannose G6: Ad-Epas1 + IL-1β (10 ng/mL) + D-mannose + Fer-1 (24 hours)	**Analysis of oxidative stress:** GSH, MDA and SOD assay kit **Cell viability assay:** CCK-8 kit **Gene quantification:** PCR **Intracellular ROS and lipid ROS level:** DCFH-DA and C11-BODIPY **Mitochondrial morphology:** Mito-Tracker **Morphology:** TB and SOFG staining **Protein expression:** WB and IF	D-mannose decreases chondrocyte ferroptosis sensitivity by inhibiting HIF-2α expression.

Ad-Epas1, adenovirus vector-endothelial PAS domain-containing protein 1; Ad-HSPA5, adenovirus vector-heat shock protein family a member 5; Ad-SND1, adenovirus vector-staphylococcal nuclease domain containing 1; AICAR, acadesine; AKT, protein kinase B; AMPKα, adenosine 5’monophosphate-activated protein kinase alpha; Annexin V-FITC/PI, annexin V‐fluorescein isothiocyanate/propidium iodide; ATX, astaxanthin; BA, BAPTA acetoxymethyl ester; BCA, biochanin A**;** CAD, cardamonin; Calcein-AM, calcein acetoxymethyl ester; CCK-8, cell counting kit-8; Co-IP, co-immunoprecipitation; Col2a1-CreERT GPx4^flox/flox^, tamoxifen-inducible chondrocyte-specific homozygous GPx4 conditional knockout; CPS, capsaicin; DCFH-DA, dichlorodihydrofluorescein diacetate assay; DFO, deferoxamine; DMT1, divalent metal transporter 1; EAAT1, excitatory amino acid transporter protein 1; ECM, extracellular matrix; EdU, 5-ethynyl-2’-deoxyuridine; ELISA, enzyme linked immunosorbent assay; EPAS1, endothelial PAS domain-containing protein 1; FAC, ferric ammonium citrate; Fer-1, ferrostatin-1; GPx, glutathione peroxidase; GSH, glutathione; GsMTx-4, M-theraphotoxin-Gr1a; GSSG, oxidised glutathione; HIF-2α, hypoxia-inducible factor 2 alpha; HO-1, heme oxygenase 1; HSPA5, heat shock protein family A member 5; IL-1β, interleukin-1β; IOOA, iron overload-induced osteoarthritis; MAPK, mitogen-activated protein kinase; MDA, malondialdehyde; MTT, methylthiazolyldiphenyl-tetrazolium bromide; NAC, N-acetyl-cysteine; NAR-L, naringenin low concentration; NAR-H, naringenin high concentration; NF-κB, nuclear factor kappa-B; NR, not reported; Nrf2, nuclear factor–erythroid factor 2; OA, osteoarthritis; Oe-NC, negative control overexpressing vector; Oe-SREBF2, sterol regulatory element binding transcription factor 2 overexpressing vector; PCR, polymerase chain reaction; PI, propidium iodide; PI3K, phosphoinositide 3-kinase; RIP, RNA immunoprecipitation; ROS, reactive oxygen species; sh-GPx4, short hairpin RNA-glutathione peroxidase 4; sh-NC, short hairpin RNA-negative control; sh-SLC3A1, short hairpin RNA-resolute carrier family 3 member 1; sh-SLC3A2, short hairpin RNA-solute carrier family 3 member 2; sh-SND1, short hairpin RNA-staphylococcal nuclease domain containing 1; sh-HSPA5, short hairpin RNA-heat shock protein family A member 5; si-DMT1, small interfering RNA-divalent metal transporter 1; si-NC, small interfering negative control; si-Nrf2, small interfering RNA-nuclear factor-erythroid factor 2; siRNA, small interfering ribonucleic acid; SLC3A2-1, solute carrier family 3 member 2; SLC3A2, solute carrier family 3 member 2; SLC7A11, solute carrier family 7 member 11; SND1, staphylococcal nuclease domain containing 1; SOD, superoxide dismutase; SOFG, safranin O-Fast Green; System Xc^-^, cystine-glutamate antiporter; SREBF2, sterol regulatory element binding transcription factor 2; STM, stigmasterol; TB, Toluidine blue; TBHP, tert-butyl hydroperoxide. TEM, transmission electron microscopy; TF3, theaflavin-3,3’-digallate; TNF-α, tumour necrosis factor alpha; TRPV1, transient receptor potential vanilloid 1; WB, Western blot.

**Table 3:** Characteristics of *in vivo* studies.

Author (year, Country)	Species (Age, weight, gender)	Sample size	Core study design	Drug delivery (Duration)	Outcome measures	Pivotal discovery
Gong et al. (27) (2023, China)	SD Rat (8 weeks, 280~320 g, male)	18 (n = 6 for each group)	G1: sham G2: ACLT/DMM G3: ACLT/DMM + CAD (20 μmol/week)	Intra-articular injection (8 weeks)	**Histology:** H&E and SOFG staining **Protein expression:** IHC	CAD alleviates cartilage damage in the rat OA model.
Guo et al. (29) (2022, China) (29)	C57BL/6 mice (8 weeks, NR, male)	56 (n = 8 for each group)	G1: sham G2: DMM G3: DMM + DFO (50 mg/kg) G4: IL-1β + DFO (100 mg/kg) G5: erastin (5 mg/kg) G6: erastin (5 mg/kg) + DFO (50 mg/Kg) G7: erastin (5 mg/kg) + DFO (100 mg/kg)	Intra-articular injection (8 weeks)	**Histology:** H&E and SOFG staining **Protein expression:** IHC	Intra-articular injection of DFO-enhanced collagen II expression inhibited erastin-induced articular chondrocyte death and delayed articular cartilage degradation and OA progression.
He et al. (24) (2023, China)	C57BL/6 mice (7 weeks, 20 g, male)	50 (n = 10 for each group)	G1: sham G2: ID (500 mg/kg) + DMM G3: ID (500 mg/kg) + DMM + NAC (100 mg/kg) G4: ID (500 mg/kg) + DMM + BCA (20 mg/kg) G5: ID (500 mg/kg) + DMM + BCA (40 mg/kg)	Intraperitoneal injection (10 weeks)	**Morphology:** micro-CT **Histology:** H&E, Perl’s Prussian blue and SOFG staining **Protein expression:** IHC	1. BCA can reduce iron deposition and the severity of KOA. 2. BCA protects against bone loss induced by iron overload.
Jing et al. (16) (2021, China) I	C57BL/6 mice (8 weeks, NR, male)	10 (n = 10 for each group)	G1: DMM G2: ID (500 mg/kg/week) + DMM	Intraperitoneal injection (8 weeks)	**Histology:** Perl’s Prussian blue, SOFG and TRAP staining **Morphology:** micro-CT **Protein expression:** IHC	Iron-overloaded mice exhibited greater cartilage destruction and elevated ADAMTS5 and MMP13 expression along with increased iron accumulation.
Lv M et al. (33) (2022, China)	Rat (unknown race) (NR)	30 (n = 6 for each group)	G1: control G2: sham G3: DMM G4: DMM + sh-NC G5: DMM+ sh-SND1	NR (NR)	**Histology:** H&E and SOFG staining **Malondialdehyde assay:** ELISA kit **Protein expression:** WB and IHC	Knockdown of SND1 upregulated HSPA5 and GPx4 in rat cartilage, inhibited inflammatory damage and ferroptosis and alleviated OA progression.
Lv Z et al. (35) (2022, China)	C57BL/6 mice(10 weeks, NR, male)	36 (n = 6 for each group)	G1 (WT mice): DMM G2 (WT mice): DMM + CPS (50 μmol/L×8 uL, 3 times/week) G3 (GPx4+/- mice): sham G4 (GPx4+/- mice): sham + CPS (50 μmol/L×8 μL, 3 times/week) G5 (GPx4+/- mice): DMM G6 (GPx4+/- mice): DMM + CPS (50 μmol/L×8 uL, 3 times/week)	Intraperitoneal injection (2/4/8 weeks)	**Histology:** H&E and SOFG staining **Morphology:** micro-CT **Protein expression:** IHC and IF	GPx4 mediates the anti-ferroptotic effect of TRPV1.
Miao et al. (28) (2022, China)	C57BL/6 mice (8 weeks, NR, male)	20 (n = 5 for each group)	G1: sham + AAV-shNC G2: ACLT + AAV-shNC G3: sham + AAV-shGPx4 G4: ACLT + AAV-shGPx4	Intraperitoneal injection (8 weeks)	**Histology:** H&E, SOFG staining and TEM **Iron ion detection in cartilage:** iron assay kit **Morphology:** micro-CT **Protein expression:** IHC **Tissue GPx activity:** GPx assay kit	GPx4 downregulation accelerated OA progression.
Pan et al. (21) (2022, China)	C57BL/6 mice (6 weeks, (20 ± 3) g, male)	50 (n = 10 for each group)	G1: DMM G2: ID (0.5 g/kg/week) + DMM G3: ID (0.5 g/kg/week) + DMM + NAC (100 mg/kg/day) G4: ID (0.5 g/kg/week) + DMM + NAR-L (60 mg/kg/day) G5: ID (0.5 g/kg/week) + DMM + NAR-H (120 mg/kg/day)	Gavage (12 weeks)	**Histology:** H&E and SOFG staining **Morphology:** micro-CT	NAR reduced synovitis, cartilage damage and subchondral bone proliferation in IOOA mice.
Wan et al. (25) (2023, China)	C57BL/6 mice (8 weeks, NR, male)	24 (n = 6 for each group)	G1: control G2: DMM G3: DMM + baicalein (1 mg/kg/week) G4: DMM + baicalein (1 mg/kg/week) + Fer-1 (1 mg/kg/week)	Intra-articular injection (10 weeks)	**Behavioural assessment:** Von Frey filaments **Histology:** H&E, SOFG and TB staining **Morphology:** micro-CT **Protein expression:** IHC and IF	1. Baicalein alleviates OA development by suppressing ferroptosis in a mouse DMM model. 2. AMPK/Nrf2/HO-1 mediates the chondroprotective effect of baicalein *in vivo*. 3. Baicalein attenuated OA pain.
Wang S et al. (23) (2022, China)	C57BL/6 mice (12 weeks, NR, NR)	20 (n = 10 for each group)	G1: DMM + PBS (10 uL/2 weeks) G2: DMM + GsMTx4 (200 μmol/L×10 μL/2 weeks)	Intra-articular injection (8 weeks)	**Histology:** SOFG staining **Morphology:** micro-CT **Protein expression:** IHC and IF	Suppression of Piezo1 attenuated cartilage ageing in a DMM osteoarthritis model.
Wang X et al. (22) (2022, China)	SD Rat (8 weeks, NR, male)	32 (n = 8 for each group)	G1: sham G2: DMM G3: DMM + Fer-1 (0.5 mg/kg/0.5 week) G4: DMM + ATX (20 mg/kg/0.5 week)	Intra-articular injection (8 weeks)	**Histology:** SOFG staining **Protein expression:** IHC	Intra-articular injection of Fer-1 and ATX delayed articular cartilage degradation and OA progression.
Wen et al. (34) (2023, China)	SD rat (8 weeks, 220~250 g, male)	NR (n ≥ 6 for each group)	G1: sham G2: Hulth G3: Hulth + solvent; G4: Hulth + UCPH-101(0.25 mg/mL×50 μL/2 days,5 times)	Intra-articular injection (4 weeks)	**Histology:** SOFG staining **Protein expression:** IHC	EAAT1 inhibition eliminated SenChos and alleviated cartilage degeneration.
Xu et al. (37) (2022, China)	SD rat (NR, 150~200 g, male)	25 (n = 5 for each group)	G1: sham G2: DMM G3: DMM + erastin (1 mg/kg/2 weeks) G4: DMM + erastin (1 mg/kg/2 weeks) + TF3 (1 mg/kg/2 weeks) G5: DMM + erastin (1 mg/kg/2 weeks) + DFO (1 mg/kg/2 weeks)	Intra-articular injection (6 weeks)	**Histology:** H&E and Masson’s staining **Morphology:** MRI **Protein expression:** IHC	TF3 inhibited OA progression by alleviating *in vivo* cartilage damage related to chondrocyte ferroptosis.
Yan et al. (32) (2022, China)	C57BL/6 mice (8 weeks, 20~25 g, male)	50 (n = 10 for each group)	G1: sham G2: DMM G3: DMM + Met (200 mg/kg/day) G4: erastin (15 mg/kg/week) G5: erastin (15 mg/kg/week) + Met (200 mg/kg/day)	Gavage (8 weeks)	**Histology:** H&E and SOFG staining **Morphology:** μ-CT **Protein expression:** IHC and IF	Met alleviates the pathological changes of OA by inhibiting ferroptosis in OA chondrocytes, alleviating subchondral sclerosis and reducing abnormal angiogenesis in subchondral bone in advanced OA.
Yao et al. (15) (2021, China)	C57BL/6 mice (8 weeks, NR, male)	32 (n = 8 for each group)	G1: sham G2: DMM G3: DMM + Fer-1 (0.1 mg/kg/0.5 weeks) G4: DMM + Fer-1 (1 mg/kg/0.5 weeks)	Intra-articular injection (8 weeks)	**Histology:** SOFG staining **Protein expression:** IHC and IF	Inhibition of ferroptosis by intra-articular injection of ferrostatin-1 seems to be a novel and promising option for the prevention of OA.
Zhou et al. (20) (2021, China)	C57BL/6 mice (8 weeks, NR, female)	28 (n = 7 for each group)	G1: sham G2: ACLT G3: Sham + D-mannose G4: ACLT + D-mannose	Oral administration of D-mannose in drinking water (20%) (4/8 weeks)	**Histology:** SOFG staining **Protein expression:** IF	D-mannose alleviates OA progression and cartilage degeneration in a mouse ACLT model.

AAV-shNC, adeno-associated virus-short hairpin RNA-negative control**;** AAV-shGPx4,s adeno-associated virus-short hairpin RNA-glutathione peroxidase 4; ACLT, anterior cruciate ligament transaction; ADAMTS5, A disintegrin and metalloproteinase with thrombospondin 5; AMPK, adenosine 5’monophosphate-activated protein kinase; ATX, astaxanthin; BCA, biochanin A; CAD, cardamonin; CPS, capsaicin; DFO, deferoxamine; DMM, destabilisation of medial meniscus; EAAT1, excitatory amino acid transporter protein 1; ELISA, enzyme linked immunosorbent assay; Fer-1, ferrostatin-1; GPx, glutathione peroxidase; GsMTx-4, M-theraphotoxin-Gr1a; H&E, haematoxylin-eosin; HO-1, heme oxygenase 1; HSPA5, heat shock protein family A member 5; ID, iron dextrin; IF, immunofluorescence; IHC, immunocytochemistry; IL-1β, interleukin-1β; IOOA, iron overload-induced osteoarthritis; KOA, knee osteoarthritis; Met, metformin; micro-CT, micro-computed tomography; MMP13, matrix metallopeptidase 13; MRI, magnetic resonance imaging; NAC, N-acetyl-cysteine; NAR-L, naringenin low concentration; NAR-H, naringenin high concentration; NR, not reported; Nrf2, nuclear factor-erythroid factor 2; OA, osteoarthritis; PBS, phosphate buffer solution; sh-NC, short hairpin RNA-negative control; sh-SND1, short hairpin RNA-staphylococcal nuclease domain containing 1; SND1, staphylococcal nuclease domain containing 1; SOFG, safranin O-Fast Green; TB, toluidine blue; TEM, transmission electron microscopy; TF3, theaflavin-3,3’-digallate; TRAP, tartrate-resistant acid phosphatase; TRPV1, transient receptor potential vanilloid 1; μ-CT, μ-computed tomography; WB, Western blot.

### 3.3 Risk of bias in studies

#### 3.3.1 *In vitro* studies

**Figure 3:**
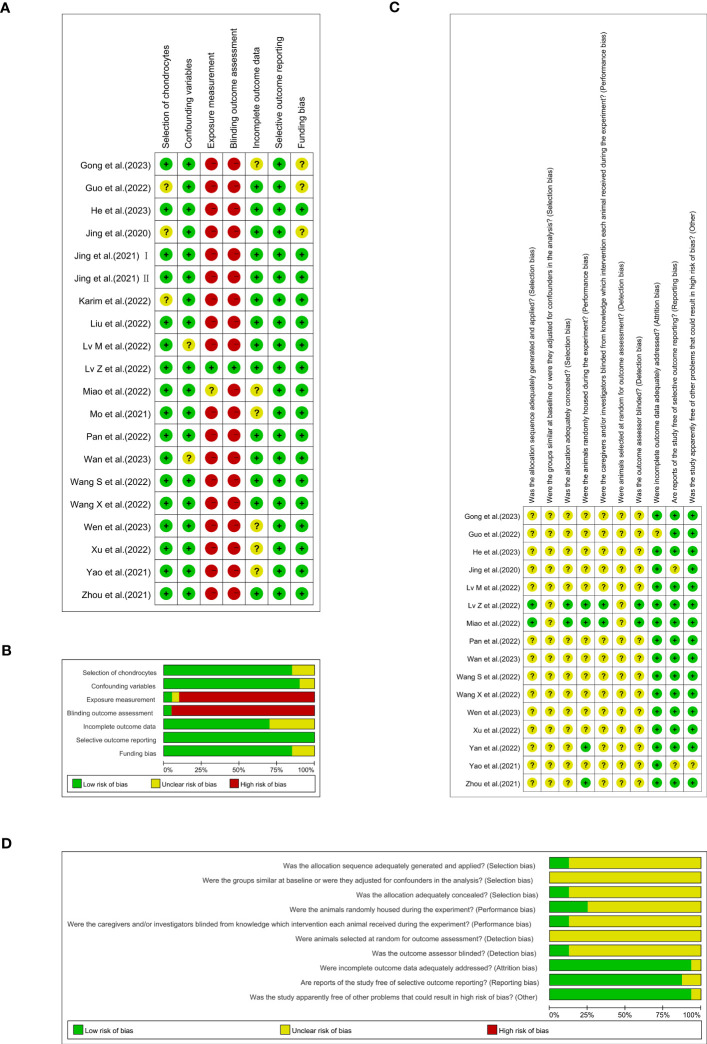
Each item’s risk of bias rating is displayed as traffic-lights and weighted bar plots: **(A)** traffic-lights plots of cellular studies, **(B)** weighted bar plots of *in vitro* studies, **(C)** traffic-lights plots of animal investigations and **(D)** weighted bar plots of *in vivo* studies.

An appraisal of risk of bias of the *in vitro* studies is shown in **Figures 3A, B**. Among these 20 studies, three (15%) were deemed to have an unclear risk of bias because they did not report whether the chondrocytes were isolated from hyaline cartilage (16, 29, 38). Just two of the studies (10%) were appraised to have an unclear risk of bias due to ‘confounding’ because they did not report the cell density or the animal characteristics from which the cells were extracted (25, 33). The bias risk of 18 studies (90%) was considered high in ‘exposure measurement’ (15, 16, 20–24, 26–31, 34–38), since these studies did not adopt two independent observers for qualitative or semi-qualitative analysis. Nineteen papers (95%) were evaluated as having a high risk in the ‘blinding outcome assessment’ domain, insomuch as the semi-quantitative analyses were not conducted blindly (15, 16, 20–31, 33, 34, 36–38). Nearly one out of three of the studies (6/20, 30%) were found to have an unclear risk of bias in ‘incomplete outcome data’ as a result of them not clearly providing information on the number of replicas (15, 27, 28, 34, 36, 37). All of the 20 studies (100%) were assessed as being at low risk of selective outcome reporting bias, since they presented the results for all the outcomes measured or for all the experimental and control groups. The ‘funding bias’ domain was appraised to possess unclear risk of bias in three of the studies (15%), since they lacked information on conflicting interests (16, 27, 29).

#### 3.3.2 *In vivo* investigations

The results of SYRCLE’s risk of bias are depicted in **Figures 3C, D**. Only two (12.5%) of 16 studies used the random-digit method and were identified as having a low risk of bias in the ‘sequence generation’ domain (28, 35). Moreover, all 16 studies (100%) were judged to have unclear risk of bias because of their absence of information regarding baseline characteristics and the random selection of animals for outcome assessment. Merely two studies (12.5%) adequately concealed the allocation of the animals during the experiment (28, 35). Twelve studies (75%) failed to completely define whether the animals were randomly housed during the experiment (15, 16, 21–25, 27, 29, 33, 34, 37). In the meantime, fourteen studies (87.5%) did not offer opportune blinding of the caregivers/investigators with respect to which intervention each animal attained during the experiment performed or if the outcome assessor was blinded (15, 16, 20–25, 27, 29, 32–34, 37). One study (6.25%) in the ‘incomplete outcome data’ domain (29) and two (12.5%) in the ‘selective outcome reporting’ domain were each reckoned to have an unclear risk of bias (15, 16). Just one experiment (6.25%) had an unclear risk of bias because it was not reflected in the domains of other bias sources (15).

### 3.4 Panorama of findings

**Figure 4:**
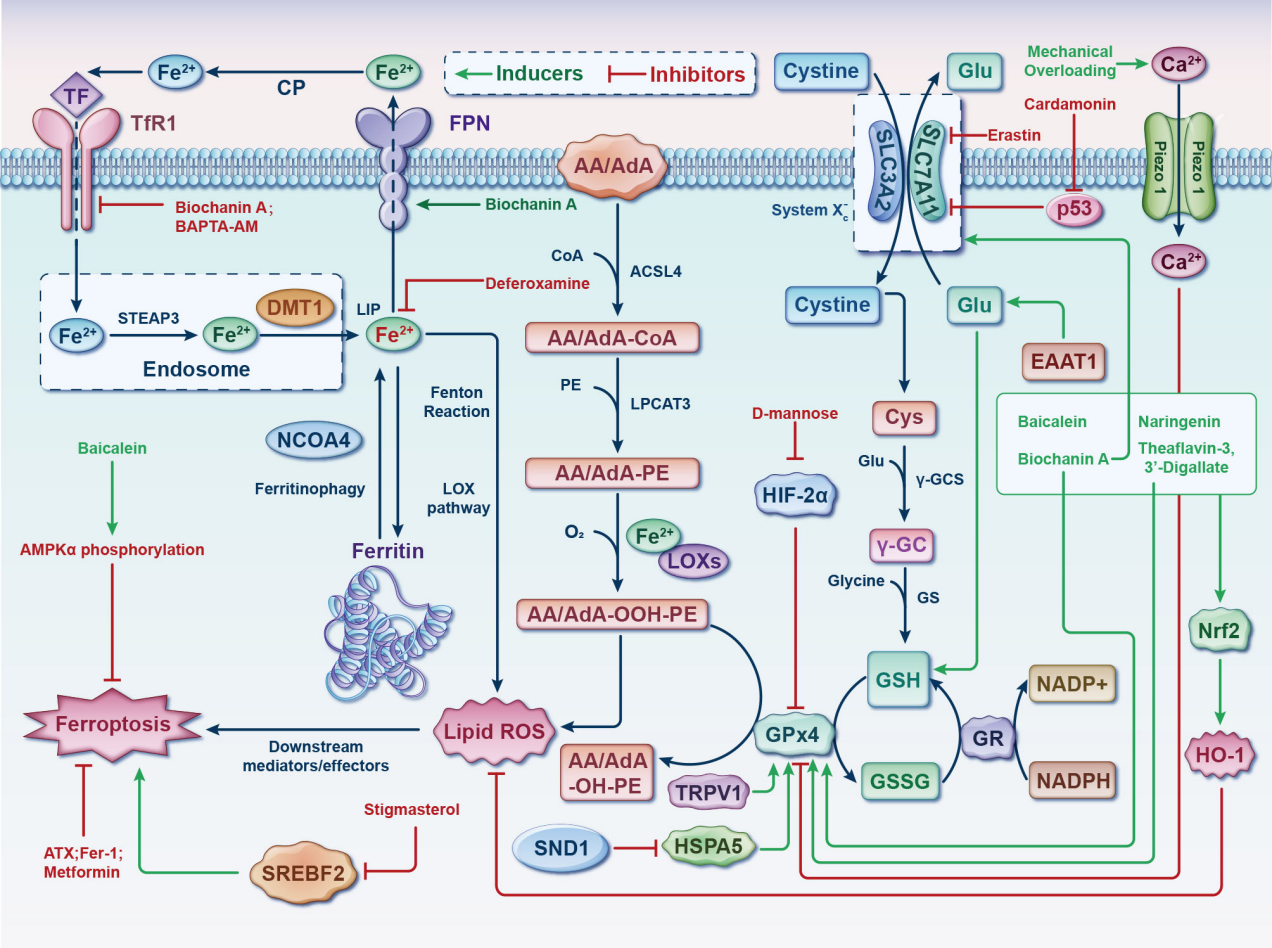
Germane molecular mechanisms of chondrocyte ferroptosis and potential therapeutic targets in osteoarthritis. The classification of ferroptotic pathways and networks can be partitioned into three fundamental clades, specifically iron metabolism, lipid metabolism, and amino acid metabolism. The transfer of inactive Fe^3+^ into chondrocytes and its subsequent reduction into Fe^2+^ within endosomes is facilitated by TFR1. Fe^2+^ is then transported to the LIP by DMT1. NCOA4 mediates ferritinophagy, which releases Fe^2+^ from ferritin through autophagic degradation. Lipid ROS is generated by Fe^2+^ via the Fenton reaction and the LOX pathway. ACSL4 is essential for the activation of polyunsaturated fatty acids, specifically AA and AdA, into AA/AdA-CoA. Subsequently, LPCAT3 facilitates the conversion of these derivatives and membrane PEs into AA/AdA-PE, which are further transformed into pro-ferroptotic lipid peroxidation through the activity of iron-containing LOXs. The process of ferroptosis is initiated through the activation of the Fenton reaction and subsequent lipid oxidation, ultimately leading to the generation of ROS within the lipid milieu. The System Xc^-^ functions as a cystine/glutamate antiporter, whereby intracellular cystine is converted to cysteine to facilitate the biosynthesis of GSH. During each catalytic cycle, GPx4 facilitates the conversion of two molecules of GSH into GSSG to effectively mitigate lipid hydroperoxide levels. Subsequently, the GSSG can be regenerated through the activity of GSH reductase in an NADPH-dependent manner. The data cut-off date of the present analysis was January 31st, 2023. AA/AdA, arachidonic acid or adrenic acid; AA/AdA-CoA, arachidonic acid or adrenic acid coenzyme A; AA/AdA-PE, arachidonic acid or adrenic acid-phosphatidylethanolamine; AA/AdA-OOH-PE, arachidonic acid or adrenic acid-hydroperoxides-phosphatidylethanolamine; AA/AdA-OH-PE, arachidonic acid or adrenic acid-hydroxides-phosphatidylethanolamine; ACSL4, acyl-CoA synthetase long-chain family member 4; AMPKα, adenosine 5’monophosphate-activated protein kinase alpha; ATX, astaxanthin; BAPTA−AM, BAPTA acetoxymethyl ester; Ca, Calcium; CoA, coenzyme A; CP, ceruloplasmin; Cys, L-cysteine; DMT1, divalent metal transporter 1; EAAT1, excitatory amino acid transporter protein 1; Fe, ferrum; Fer-1, ferrostatin-1; FPN, ferroportin; γ-GC, gamma-glutamylcysteine; γ-GCS, gamma-glutamylcysteine synthetase; Glu, L-glutamate; GPx4, glutathione peroxidase 4; GR, glutathione reductase; GS, glutathione synthetase; GSH, glutathione; GSSG, di-glutathione; HIF-2α, hypoxia-inducible factor 2 alpha; HO-1, heme oxygenase 1; HSPA5, heat shock protein family A member 5; LIP, labile iron pool; Lip-1, liproxstatin-1; LOX, lipoxygenase; LPCAT3, lysophosphatidylcholine acyltransferase 3; NADP^+^, nicotinamide-adenine dinucleotide phosphate; NADPH, reduced form of nicotinamide-adenine dinucleotide phosphate; NCOA4, nuclear receptor coactivator 4; Nrf2, nuclear factor-erythroid factor 2; PE, phosphatidylethanolamine; ROS, reactive oxygen species; SLC3A2, solute carrier family 3 member 2; SLC7A11, solute carrier family 7 member 11; SND1, staphylococcal nuclease domain containing 1; SREBF2, sterol regulatory element binding transcription factor 2; STEAP3, 6-transmembrane epithelial antigen of the prostate 3; TF, transferrin; TFR1, transferrin receptor 1; TRPV1, transient receptor potential vanilloid 1.

Ferroptosis involved in the pathogenesis of OA have demonstrated that ferroptosis could be a potential target for the treatment of it. Both OA and ferroptosis pertain to intricate pathways that are still not entirely understood. Further research is required to utterly delve into the role of these processes and identify potential interventions to target them with the objective of prevention or remedy. The discoveries of this present up-to-date SR are based on a synthesis and evaluation of the existing 21 studies mainly focuses on two of the three elements of Ferroptosis: iron homeostasis disorder and glutathione peroxidase 4 (GPx4) activity loss. However, the lipid peroxidation caused by polyunsaturated fatty acids need to be further explored (**Figure 4**).

From this, chondrocyte ferroptosis acts as a pivotal initiator of OA. The results of the preclinical studies mentioned above have authenticated that the targeted ferroptosis of chondrocytes holds enormous potential for clinical applications and is paramount for a ‘precision medicine’ approach to the clinical management of OA. During this process, not only do the molecular mechanisms of chondrocyte ferroptosis demand further refinement to identify more treatment targets but the research of ferroptosis inhibitors, the drug delivery system and ferroptotic detection methods are also required to closely meet the development needs.

### 3.5 Regulation of systemic and cellular iron metabolism in the cartilage

In view of abnormal iron metabolism being one of chief features of ferroptosis, six of 20 papers (30%) were focused on iron overload–induced osteoarthritis (IOOA) and ferroptosis. One research team from Shandong, China, found iron to be involved in the progression of OA, that iron-overloaded mice exhibited greater enhanced cartilage catabolism (16) and that abating iron influx by inhibiting divalent metal transporter 1 (DMT1) activity might be an appealing therapeutic target for OA remedy. The inhibition of DMT1 suppressed interleukin-1β (IL-1β)–induced inflammatory response and ECM degradation via the blockade of mitogen-activated protein kinase (MAPK) and phosphoinositide 3-kinase (PI3K)/protein kinase B (AKT)/nuclear factor kappa-B (NF-κB) pathways. In the same year, the authors followed up with a study revealing that pro-inflammatory cytokines possess the capability to wreck chondrocytes’ iron homeostasis, propelling an iron influx (30). Iron overload–induced oxidative stress and mitochondrial dysfunction are of crucial importance in iron overload–evoked cartilage degeneration. Again in 2021, a third paper by Jing et al. went on to point out that calcium chelators may be of value in the treatment of IOOA advancement (26). One kind of calcium chelator in their research, BAPTA acetoxymethyl ester (BAPTA−AM), was capable of counterbalancing iron overload–engendered chondrocyte mitochondrial malfunction and chondral deterioration. Thereafter, a study by Karim et al. showed that dysregulated iron metabolism retards cellular iron homeostasis, which compromises the functional integrity of chondrocytes and leads to oxidative stress and apoptosis (38).

The ferroptosis inhibition effect of deferoxamine (DFO) in OA was verified by Miao et al. in February 2022 (28). Their *in-vivo* experiments suggested that DFO and Fer-1 could play a protective role in OA progression by inhibiting ferroptosis. After approximately one month, this outcome was supported partially by another Chinese study team (29). DFO alleviated the inflammatory response and ECM degradation in chondrocytes induced by IL-1β by inhibiting chondrocyte ferroptosis. Nrf2 signalling mediated the protective effects of DFO on chondrocytes induced by IL-1β. Intra-articular injection of DFO enhanced collagen II expression, inhibited erastin-induced articular chondrocyte death and delayed articular cartilage degradation and OA progression. Apart from DFO (15), there is a variety of alternative iron chelators when focusing on IOOA. Deferiprone, an effective oral iron chelator currently approved by the U.S. Food and Drug Administration (FDA), can easily pass through the cell membrane and efficiently chelate intracellular iron because of its small molecular weight and lipophilic nature (39). Deferasirox (40), another FDA-authorised iron chelator, has efficacy in OA that remains much to be desired. In addition to these, it is also worth trying to employ dexrazoxane and cyclipirox (12). Of note, a combination of iron chelators is better at removing non‐protein-bound or free iron than one drug alone (41).

In addition to the iron chelation strategy mentioned earlier, Chinese herbal medicine seems to be a good choice for IOOA. In July 2022, one Traditional Chinese Medicine research team revealed that naringenin (NAR) can ease oxidative stress through the nuclear factor–erythroid factor 2 (Nrf2)/heme oxygenase 1 (HO-1) pathway and alleviate cartilage damage under excess iron deposits, which has the potential to cure IOOA (21).

### 3.6 Activation of the System Xc^−^/GSH/GPx4 Axis

By generating a stable gene knockdown chondrocyte model, Miao et al. in early 2022 attested that GPx4 downregulation can increase the sensitivity of chondrocytes to oxidative stress, aggravate ECM degradation through the MAPK/NF-κB pathway and subsequently expedite OA progression (28). The staphylococcal nuclease domain containing 1 (SND1) protein was reported in March 2022 to facilitate the degradation of GPx4 by destabilising heat shock protein family A member 5 (HSPA5) mRNA and suppressing HSPA5 expression, promoting ferroptosis in OA chondrocytes (33). As a part of cystine-glutamate antiporter (System Xc^-^), solute carrier family 3 member 2 (SLC3A2) was shown to be a potential therapeutic target of OA involved in ferroptosis by integrating bioinformatics and experiments in October of the same year (31). After a month, single-cell RNA sequencing analysis revealed transient receptor potential vanilloid 1 (TRPV1) as an anti-ferroptotic target in chondrocytes that abrogated ferroptosis by promoting GPx4 expression (35). The underlying mechanism of mechanical overloading in chondrocytes was described in late 2022 (23). Through Piezo1 channel-mediated calcium influx, mechanical overload induced GPx4-regulated chondrocyte ferroptosis in OA. At the beginning of 2023, the mechanism of ferroptosis resistance in senescent chondrocytes (SenChos) was explored. The excitatory amino acid transporter protein 1 (EAAT1)-glutamate-GPx4 anti-ferroptosis axis was recognised as a critical determinant of SenChos survival (34). Therefore, EAAT1 shows promise to emerge as an efficacious and specific remedial target in OA. He et al. reported that disordered iron metabolism can suppress the expression of collagen II and induce matrix metallopeptidase (MMP) expression by catalysing ROS generation, while biochanin A (BCA) is capable of defending against OA by modulating iron levels and the Nrf2/System Xc^-^/GPx4 axis (24).

GPx4 is a critical intracellular negative regulator of lipid peroxidation. It utilises glutathione (GSH) to catalyse the conversion of hazardous lipid peroxide into harmless lipid hydroxy and subsequently prevents cells from ferroptosis caused by lipid peroxidation. With the continuous progress made in the investigation of chondrocyte ferroptosis, an increasing amount of evidence has validated that GPx4 is one master chaperon of chondrocyte ferroptosis. Eight out of 21 studies in the present SR involved this key regulator. Thus, targeting GPx4 (e.g. dopamine (42, 43), carvacrol (44) and selenium (45)) to modulate chondrocyte ferroptosis may have therapeutic value in the prevention and treatment of OA. What’s more, as the central repressor of ferroptosis, GPx4’s activity hinges on GSH manufactured from the activation of System Xc^-^ (46).

### 3.7 Detection means of chondrocyte ferroptosis

#### 3.7.1 Existing means

In contrast to other types of programmed cell death (PCD) that have been investigated relatively thoroughly, there is no standardised approach for the detection of ferroptosis. As recapitulated in **Tables 2**, **3**, the most frequently applied methods for detecting ferroptosis include transmission electron microscopy and fluorescent dye to observe the morphology of chondrocytes and organelles (particularly mitochondrion) and detect chondrocytes’ viability/toxicity and cellular and histrionic iron levels, lipid peroxidation levels, mitochondrial membrane potential and ferroptosis-related gene expression at both the nucleic acid and protein levels. However, manifold issues remain. At present, the ferroptotic results that have been obtained are mostly descriptive, while it is also possible that other forms of PCD exhibit similar characteristics as ferroptosis. Consequently, the interpretation of these results requires even greater caution. For pre-clinical research, the most critical issue is who is the ultimate executor that enables ferroptosis to occur after lipid peroxidation, which might also contribute to the discovery of additional hallmarks of ferroptosis and significantly differentiate ferroptosis from other forms of PCD. Molecular mechanisms underlying chondrocyte ferroptosis in OA urgently require in-depth investigation to offer ideal and determinant biomarkers.

#### 3.7.2 Burgeoning methodology

Deciphering gene functions is consequential to apprehend the signalling cascades and pathways that administrate senescence and ferroptosis. Modern medicine is striding forward into a new epoch in which advanced and highly integrative functional annotation strategies are being developed to elucidate the functions of all human genes. As a result of advances made in high-throughput technologies, there is a clear trend towards adopting omics analysis in biomedical research to help expound the knotty nexus between molecular layers (47). However, there is a complex crosstalk between different molecules that may have been overlooked by single-omics studies (48). Disease development and clinical presentation can be affected by cross-omics interactions (49, 50). A correct verdict can only be reached when diverse assays are integrated in a thorough manner. In the era of precision medicine, multi-omics is an emerging analytical methodology that is expected to provide new insights into the mechanisms involved in disease (48, 51).

**Figure 5:**
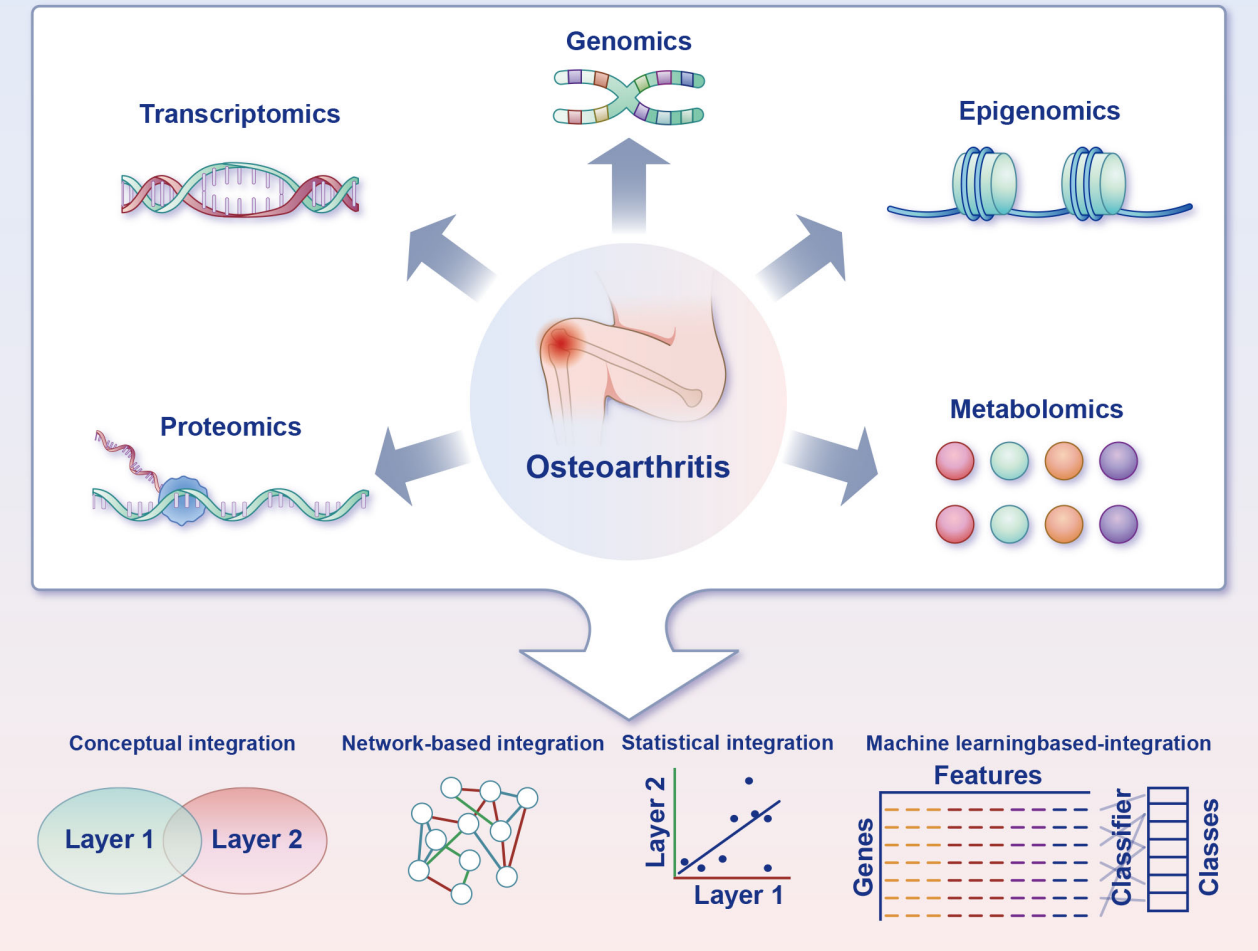
Multi-omics strategies for charting the ferroptosis of chondrocytes in osteoarthritis. Acquisition of multi-omics data can be accomplished through experimental techniques or by accessing functional genomics databases. Transcriptomics is frequently a pivotal component of the research design, and the inclusion of supplementary omics layers can reveal complementary facets of gene function. Furthermore, distinct combinations of omics layers can be employed to address particularly biological inquiries. Multiple integration approaches are available to leverage the information gleaned from the diverse omics layers and facilitate the formulation of novel, verifiable hypotheses pertaining to biological pathways and processes.

Data from multiple omics sources, such as transcriptomics, proteomics and metabolomics, can be integrated to unveil the involute working of systems biology employing machine learning–based predictive algorithms (52). Machine learning–based integration furnishes methods to analyse the various omics data, guaranteeing the discovery of new biomarkers (52). These biomarkers will have the potential to decipher the mechanism of chondrocyte ferroptosis in OA, plumb novel treatment targets and achieve predictive, preventive and personalised medicine at length (**Figure 5**).

### 3.8 Pharmaceuticals for targeting ferroptotic factors and drug delivery system

#### 3.8.1 Discovery of incorporated literature

In the last 10 years, research on ferroptosis inhibitors has attracted the attention of scientists throughout the world and is one of the current hotspots in the domain of OA investigations. This up-to-date SR includes a total of nine studies focusing on this topic and relevant research. As the earliest investigators of chondrocyte ferroptosis, Yao et al. asserted at the beginning of 2021 that intra-articular injection of ferrostatin-1 (Fer-1) is an encouraging alternative for the prophylaxis of OA. In September 2021, D-mannose was confirmed as being capable of having a chondroprotective effect by attenuating the sensitivity of chondrocytes to ferroptosis and alleviating OA progression by inhibiting hypoxia-inducible factor 2 alpha (HIF-2α) expression (20). Also in late 2021, Mo et al. successfully utilised stigmasterol (STM) to mitigate chondrocyte injury induced by IL-1β by regulating ferroptosis by down-regulating sterol regulatory element binding transcription factor 2 (SREBF2) (36).

In June 2022, it was reported that the classic hypoglycaemic agent metformin is capable of assuaging the pathological changes of OA by inhibiting ferroptosis in OA chondrocytes (32). Merely two months later, Wang et al. affirmed that both Fer-1 and astaxanthin (ATX) have the capacity to ease chondrocyte injury and OA progression by inhibiting ferroptosis and regulating mitochondrial function (22). These results are partly consistent with the view put forth by Yao et al. one year earlier (15). The function of Theaflavin-3,3’-digallate (TF3) in inhibiting OA progression by alleviating cartilage damage related to chondrocyte ferroptosis was identified in the late 2022 by Xu et al. (37). The inhibitory effects of TF3 on chondrocyte ferroptosis are mediated through activation of Nrf2/GPx4 signalling.

Two related papers on the inhibitors of chondrocyte ferroptosis have been published since the start of 2023. Wan et al. found that baicalein alleviated OA development *in vivo* and *in vitro* by suppressing chondrocyte ferroptosis by improving the activity of adenosine 5’monophosphate-activated protein kinase (AMPK)/Nrf2/HO-1 signalling (25), while the study performed by Gong et al. certified that intra-articular injection of cardamonin (CAD) significantly ameliorates cartilage damage by inhibiting ferroptosis via the p53/solute carrier family 7 member 11 (SLC7A11)/GPx4 signalling pathway (27).

#### 3.8.2 Future vistas

As previously stated, the primary emphasis in pharmaceutical research pertaining to chondrocytes’ ferroptosis centers on the regulation of the two principal components of the ferroptotic network, namely oxidative stress and body iron homeostasis, with minimal attention directed towards lipid peroxidation. The key to ferroptosis is a group of tailored polyunsaturated fatty acid–containing phospholipids. The corresponding lipid peroxides and peroxyl radicals are the execution molecules of ferroptosis. Manipulating lipid peroxidation to suppress chondrocyte ferroptosis can be regarded as an avenue for OA treatment. Acyl-CoA synthetase long-chain family member 4 (ACSL4), the first identified pro-ferroptotic gene product (13), is a member of the ACSL family. Different from other family members, ACSL4 can catalyse arachidonic acid (AA) to synthesise arachidonoyl coenzyme A and then participate in the synthesis of phosphatidylethanolamine (PE). As the main component of phospholipids in cell membranes, PE occupies a significant place in the lipid peroxidation of ferroptosis. Doll et al. confirmed that ACSL4 knockout can significantly inhibit the esterification of AA into PE (53), thereby reducing the susceptibility of cells to ferroptosis and preventing the occurrence of it. As the selective inhibitors of ACSL4, thiazolidinediones (TZDs, e.g. rosiglitazone, pioglitazone and troglitazone) are supposed to restrain OA progression.

It is noteworthy that TZDs is a category of anti-diabetic medications that elicit insulin sensitization in adipocytes by means of activating the peroxisome proliferator-activated receptor-gamma. While OA is one type of the age-correlated joint and bone disorders that are commonly seen in middle-aged and elderly adults (1). These patients also tend to suffer from other chronic diseases, such as diabetes mellitus and cardiovascular and cerebrovascular diseases. From this, we were drawn to consider filtering pharmaceuticals with anti-ferroptosis from existing medicines to fulfil the purpose of ‘one drug, multiple illnesses’. Mishima et al. identified various FDA-approved drugs and hormones with anti-ferroptotic properties (54), including rifampicin, promethazine, omeprazole, indole-3-carbinol, carvedilol, propranolol, oestradiol and thyroid hormones. The anti-ferroptotic drug effects were closely associated with the scavenging of lipid peroxyl radicals (54). As a free-radical scavenger, edaravone is thought to reduce oxidative stress and has been used in patients with cerebral infarction as a support therapy for stroke (55). Besides, it can protect against ferroptosis *in vitro*, as was demonstrated in 2019 by Homma et al (56). Bazedoxifene, a kind of FDA-ratified selective oestrogen‐receptor modulator, has been used to prevent and treat postmenopausal osteoporosis (57). Conlon et al. reported in 2021 that bazedoxifene acted as a potent radical-trapping antioxidant inhibitor of ferroptosis both *in vitro* and *in vivo (* 58). In addition to these findings, it has been affirmed that the hypocholesterolaemic drug probucol and its analogues suppress ferroptosis (59).

Apart from the diverse synthetic chemical drugs sanctioned for commercialization by the FDA as previously stated, natural products, especially those from plants, have been indispensable sources of medication discovery for decades. Many plants’ secondary metabolites, such as polyphenols, are increasingly favoured. Due to the structural characteristics of natural compounds, most of them have intrinsic antioxidant activity. Some of them have been confirmed to act as free radical scavengers and lipid peroxidation inhibitors, and thus impede ferroptosis. These natural compounds include quercetin (60), puerarin (61), kaempferide (62), kaempferol (62), gastrodin (63), curcumin (64, 65) and glycyrrhizin (66). There is no denying that such agents will provide invaluable benefits for the treatment of OA.

**Figure 6:**
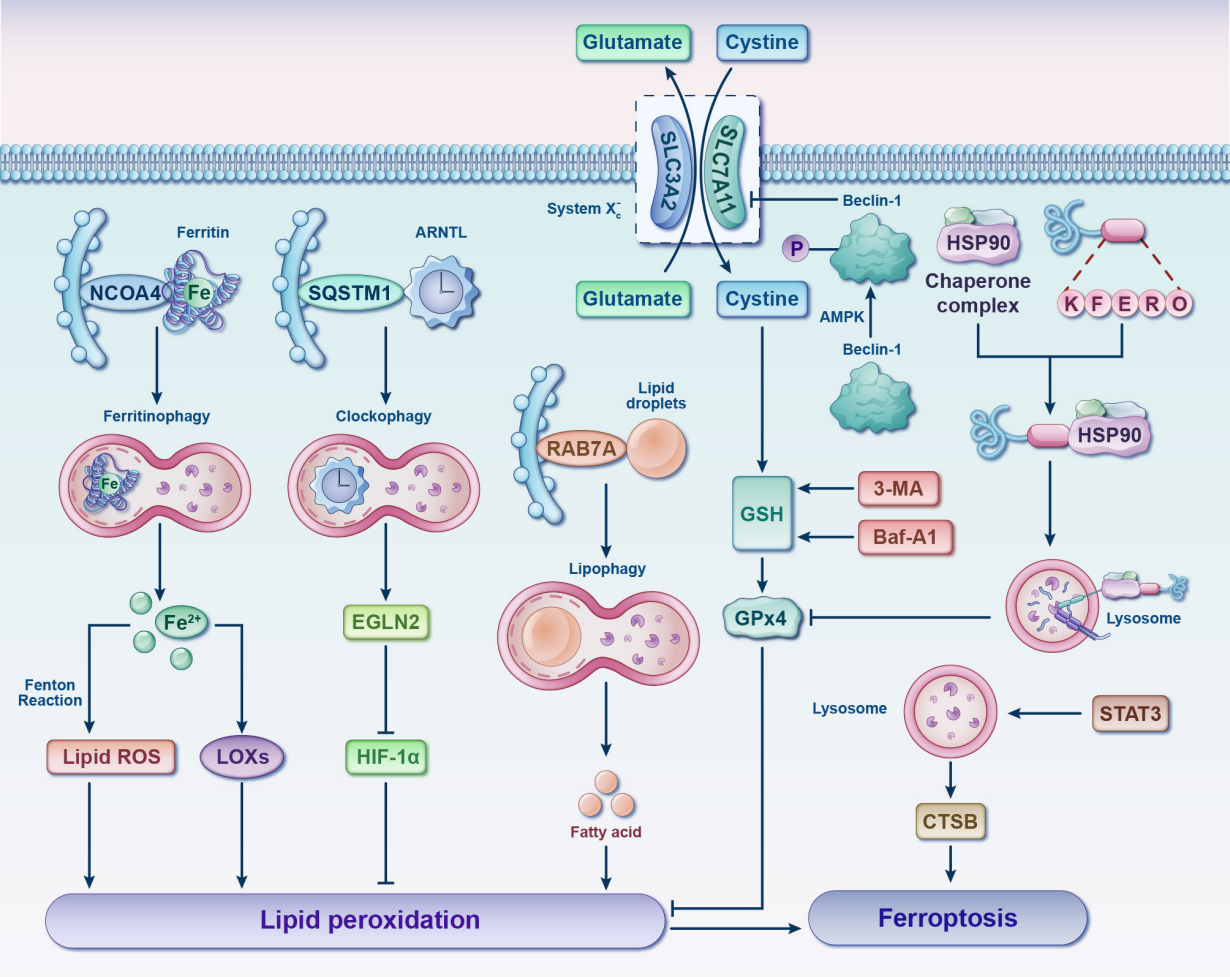
Interplay between ferroptosis and autophagy. Various forms of selective autophagy, such as ferritinophagy, lipophagy, clockophagy, and CMA, induce lipid peroxidation and ferroptosis by means of promoting the degradation of ferritin, lipid droplets, ARNTL, and GPx4, respectively. Following phosphorylation by AMPK, Beclin-1 facilitates ferroptosis by binding to and inhibiting the activity of System Xc^-^. Autophagy inhibitors, such as 3-MA and Baf-A1, can impede GSH depletion-dependent ferroptosis. Additionally, STAT3-mediated CTSB expression and lysosomal cell death contribute to the promotion of ferroptosis. 3-MA, 3-methyladenine; AMPK, adenosine 5’monophosphate-activated protein kinase; ARNTL, aryl hydrocarbon receptor nuclear translocator-like protein 1; Baf-A1, bafilomycin A1; CTSB, cathepsin B; EGLN2, egl-9 family hypoxia inducible factor 2; Fe, ferrum; GPx4, glutathione peroxidase 4; GSH, glutathione; HIF-1α, hypoxia-inducible factor 1 alpha; HSP90, heat shock protein 90; LOXs, lipoxygenases; NCOA4, nuclear receptor coactivator 4; RAB7A, RAS-related protein Rab-7a; ROS, reactive oxygen species; SQSTM1, sequestosome 1; STAT3, signal transducer and activator of transcription 3.

Despite being distinct from other forms of cell death, it is worth noting that the significant interplay between autophagy and ferroptosis has captured growing attention in recent years (**Figure 6**) (67–70). Such crosstalk might shed important novel light on pharmaceutical research and development of chondrocytes’ ferroptosis inhibitors. Oxidative stress and lipid peroxidation products (such as malondialdehyde, ROS, and 4-hydroxynonenal) are powerful inducers of autophagy, while excessive autophagy promotes ferroptosis (68, 69). Typically, ferritinophagy, lipophagy, clockophagy and chaperone-mediated autophagy (CMA) facilitate cell predisposition to ferroptosis by degrading ferritin, lipid droplets, aryl hydrocarbon receptor nuclear translocator-like protein 1 (ARNTL) and GPx4, respectively (71). Critical regulators of autophagy such as beclin-1 (72, 73), and high mobility group box 1 (HMGB 1) (74, 75) will consequently also have an impact on ferroptosis. Further investigation is required to explore the interplay between ferroptosis and autophagy in the context of oxidative stress, with a view to identify potential targets for synergistic combination therapy aimed at achieving “one drug-multiple targets-OA” for future interventions.

Furthermore, there remains a dearth of research on the optimal parameters for the utilization of chondrocytes’ ferroptosis inhibitors, including the conditions of application, time point of onset, dosage, form of administration, and duration of efficacy. Majority of extant studies investigating the pathological effects of ferroptosis have been conducted in animal models and specific cell types, with limited assessment of its clinical safety and efficacy. Thus, further pre-clinical and clinical trials are warranted to elucidate the role of ferroptosis in the human body and establish a foundation for the development of therapeutic agents for the treatment of human diseases.

#### 3.8.3 Novel drug delivery system of OA

**Figure 7:**
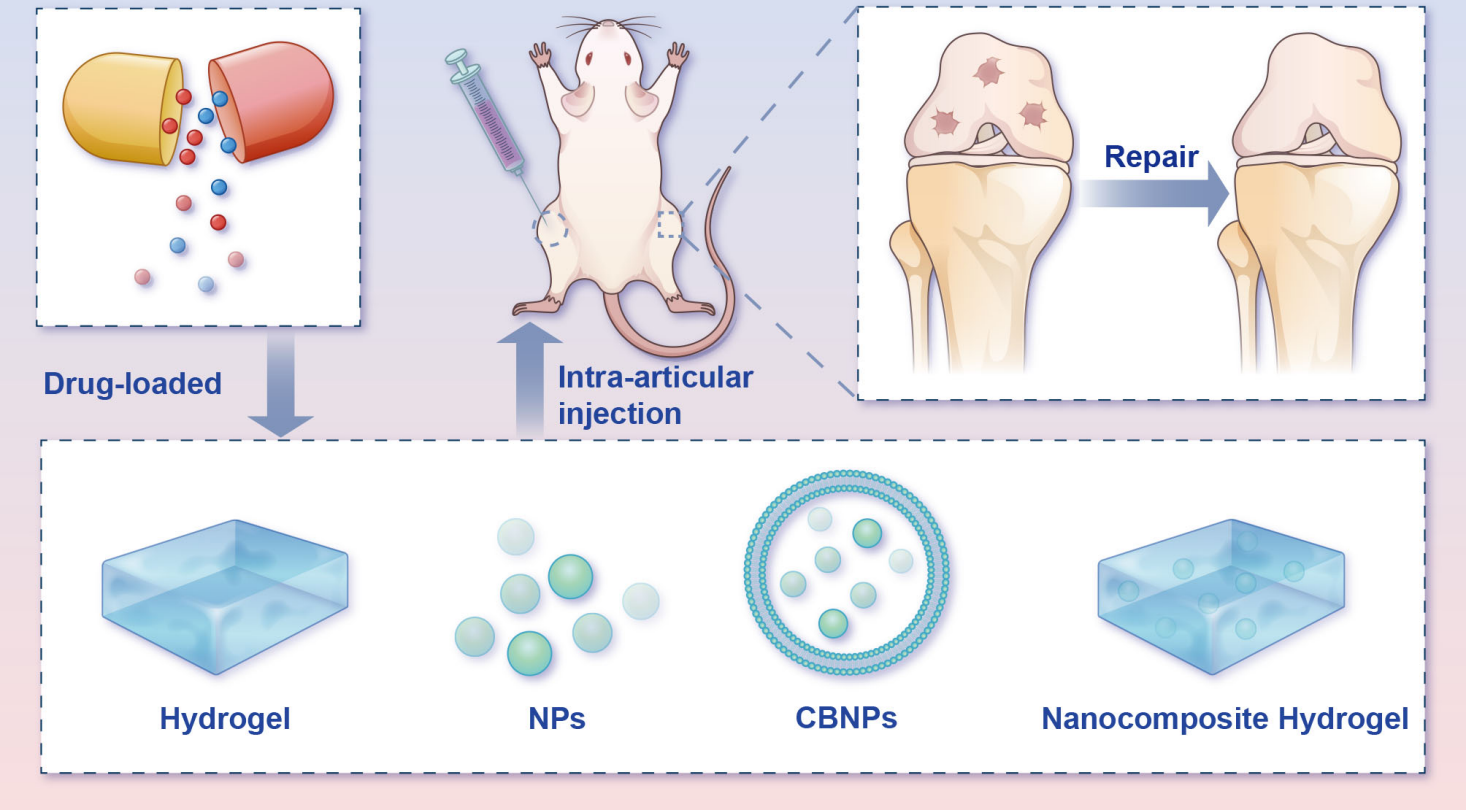
Potential intra-articular administration in the remedy of osteoarthritis animal models. Local administration of pharmaceuticals assuages the progression of OA in the *in vivo* model. Bioactive materials (e.g., hydrogels, NPs, CBNPs, and nanocomposite hydrogels) loaded with drugs mimic the ECM microstructure and improve medicine-release properties, therefore advancing OA rehabilitation and rebuilding efficacy. CBNPs, cell membrane biomimetic nanoparticles; NPs, nanoparticles.

As indicated in **Table 3**, there are several means of administration for *in vivo* experiments (intra-articular injection, gavage, intra-peritoneal injection and oral administration of drugs in drinking water (20%)). However, orally administered pharmaceuticals are more often than not impeded by side effects (e.g. gastrointestinal symptoms). Considering the closed structure of joints and the limited vascularity of articular cartilage *in vivo*, intra-articular injection seems to be superior to the effect of oral or intra-peritoneal injection for remedying OA (76), and small molecules are easily cleared by the lymphatic system and blood vessels after being injected into a joint cavity (77, 78). A high clearance rate will inevitably entail a drug’s failure to reach its remedial dose. For effective therapy, multiple and frequent administrations are required. Multiple injections of fluid into a joint may increase the risk of inadvertent joint infection, which greatly curbs this type of administration. To date, more than 70% of the current market drugs and recently discovered drugs have been found to have poor water solubility (79). In the foreseeable future, a major challenge for the pharmaceutical industry will be to enhance the solubility of active pharmaceutical ingredients (80). To release drugs controllably, enhance the half-life of drugs and promote the repair of cartilage injury, it is necessary to develop novel sustained-release drug delivery systems (DDS) for OA (**Figure 7**).

Hydrogels, one kind of three-dimensional networks of crosslinked hydrophilic polymer with good biocompatibility, have for years been widely used for biomedical applications (81–83). However, before hydrogels can see widespread use and clinical translation, several deficiencies must be to be addressed, including their weak mechanical properties and compromised bioactivity (84). The great potential of nanomedicine for cartilage repair has brought a new dawn to cartilage tissue engineering. Nanocomposite hydrogels can markedly mimic natural cartilage components with excellent histocompatibility, exhibiting unique biological effects (85). In view of this, the number of investigations on nanocomposite hydrogels has risen steeply. A combination of nanoparticles (NPs) and hydrogel offers a variety of synergistic properties superior to their individual ones, and in the meantime, the rehabilitative capacity of cartilage has apparently been boosted (86, 87). In addition to nanocomposite hydrogels, NPs themselves have also been widely investigated for the treatment of bone-related diseases due to their special characteristics (88). The application of NPs for DDS can not only prolong the *in vivo* retention time of drugs but also bolster the biodistribution and fulfil the aim of passive and active targeting at the diseased site (88, 89).

More recently, cell membrane biomimetic nanoparticles (CBNPs) have received extensive attention, since they offer superb biocompatibility and low immunogenicity (90). These CBNPs consist of an NP core and wrapped natural cell membranes on the periphery. CBNPs integrate the immanent superiority of cells that served as the membrane source, with the multifunctional nanomaterial in the core, which provides stronger drug-loaded capability, longer retention time and immune escape ability (91). Different cell types endow the diverse cell membranes with various biological properties. For homologous targeting in OA, chondrocytes’ membrane biomimetic nanoparticles are expected to become stimuli-responsive carriers that can be released under specific pathophysiological states.

### 3.9 Limitations

This SR has several limitations. The first limitation is that the studies it includes were all carried out in cellular or animal models, which hardly mirror the proceedings occurring within humanity. Secondly, the studies embraced manifold designs and approaches, which may make it challenging to compare their results. It is rewarding to note that the mechanisms latent in the link connecting chondrocytes, ferroptosis and OA are likely sophisticated and multi-dimensional. Thirdly, the quality assessment using the risk of bias tool found that critical details regarding the design and conduct of the included experiments were missing. Accordingly, most studies were unable to estimate the risk of bias. As a major concern, the absence of vital methodological details may indicate neglect in using these methods, potentially inducing skewed results (92). Moreover, it is likely that pertinent studies may still have been omitted, although seven cardinal electronic databases were employed to identify potential studies. Given the circumscribed size of the existing study, follow-up in-depth investigations are exceedingly worthy and pressing. In addition, nearly all studies (20/21, 95.23%) originated from China, leading to possibly poor extrapolation of the findings. Finally, the inclusion of the published studies and the exclusion of non-English studies undeniably renders selection bias.

## 4 Conclusion

As described previously, the pathogenesis of OA is complex, and existing treatments that target its symptoms are often insufficient. As a result, exploring the pathogenesis and seeking new targets have formed the new breakthrough point for the prevention and control of OA. Gratifyingly, there is copious evidence for ferroptosis to be set as a promising therapeutic target for disease-modifying interventions in OA. Moving forward, there remain pressing challenges and inquiries that require attention. Current knowledge of chondrocyte ferroptosis in OA is possibly only the tip of the iceberg, and investigations pertaining to it are still in their infancy. In terms of pre-clinical investigation, the crux is identifying the definitive agent responsible for facilitating ferroptosis subsequent to lipid peroxidation, as well as determining the optimal biomarker for the prevention or prognosis of OA.

Accordingly, comprehending the network organization of the ferroptosis system, as opposed to the impact of individual regulators, assumes greater significance in gaining a profound understanding of the mechanisms that underlie ferroptosis. Elucidating the ferroptotic network will also furnish valuable insights into the diagnosis and treatment of OA. Therefore, additional research is necessary to uncover the latent mechanisms that underlie ferroptosis in the onset and progression of OA. It is anticipated that more efficacious and suitable strategies for treatment and prophylaxis will emerge in the foreseeable future.

## Data availability statement

The original contributions presented in the study are included in the article/**Supplementary Material**. Further inquiries can be directed to the corresponding authors.

## Author contributions

SC: Conceptualisation, data analysis and manuscript writing. YW: Data curation, investigation and manuscript writing. FY, SL, and HX: Data curation, reviewing and editing. TQ and JW: Methodology, software and supervision. AX, PL, and HZ: Project administration and funding acquisition. All authors contributed to the article and approved the submitted version.
